# Pulmonary pharmacokinetics and lesion penetration of antimicrobials in patients with nontuberculous mycobacterial disease

**DOI:** 10.1128/aac.00820-26

**Published:** 2026-10-01

**Authors:** Fumiya Watanabe, Miyako Hiramatsu, Toru Kawakami, Teruaki Oka, Hayato Nanami, Kiyomi Shimoda, Kazuhiko Hanada, Yuji Shiraishi, Kozo Morimoto

**Affiliations:** 1 Department of Pharmacometrics and Pharmacokinetics, Meiji Pharmaceutical University, Tokyo, Japan; 2 Department of Pharmacy, Fukujuji Hospital, Japan Anti-Tuberculosis Association, Tokyo, Japan; 3 Department of Thoracic Surgery, Respiratory Disease Center, Fukujuji Hospital, Japan Anti-Tuberculosis Association, Tokyo, Japan; 4 Department of Thoracic Surgery, Higashi-yamato Hospital 469425 , Tokyo, Japan; 5 Department of Pathology, Respiratory Disease Center, Fukujuji Hospital, Japan Anti-Tuberculosis Association, Tokyo, Japan; 6 Department of Respiratory Medicine, Respiratory Disease Centre, Fukujuji Hospital, Japan Anti-Tuberculosis Association, Tokyo, Japan; 7 Department of Clinical Research, Fukujuji Hospital, Japan Anti-Tuberculosis Association, Tokyo, Japan; 8 Department of Clinical Mycobacteriosis, Nagasaki University Graduate School of Biomedical Sciences, Nagasaki, Japan; Queen Mary University of London, London, United Kingdom

**Keywords:** nontuberculous mycobacteria, penetration, pulmonary lesions, pharmacokinetics, *Mycobacterium avium*

## Abstract

The intrapulmonary pharmacokinetics of antimicrobial agents used to treat nontuberculous mycobacterial (NTM) pulmonary disease remains poorly characterized, limiting the optimization of dosing regimens. This study characterized the plasma and intrapulmonary pharmacokinetics of azithromycin, ethambutol, rifampicin, clofazimine, and amikacin, as well as their penetration into pulmonary lesion sites. We prospectively enrolled patients undergoing guideline-based treatment for NTM pulmonary disease who were indicated for surgical resection at a single center in Japan. Drug concentrations were measured in the plasma and lung samples and analyzed using a population pharmacokinetic model. The lung lesion site, cavity, or nodule/bronchiectatic lesion was evaluated as covariates of the lung-to-plasma partition ratios. Twenty-four patients were enrolled in the study. The estimated lung-to-plasma partition coefficients and intrapulmonary half-lives were 535-fold and 6.3 months for azithromycin, 40-fold and 2.0 months for ethambutol, 5.2-fold and 6.3 min for rifampicin, 18,800-fold and 22 years for clofazimine, 1.3-fold and 2.3 h for intravenous amikacin, and 1.3-fold and 5.0 h for inhaled amikacin, respectively. Evaluation of drug penetration into cavities and nodular/bronchiectatic lesions showed no clearly reduced concentration compared to a normal lung tissue. The intrapulmonary pharmacokinetic characteristics identified in this study provide valuable information for optimizing antimicrobial dosing regimens in the treatment of NTM pulmonary disease. Furthermore, the absence of clearly reduced drug penetration into lesion sites suggests that lesion phenotype alone may have limited value in guiding antimicrobial selection.

## INTRODUCTION

The incidence and prevalence of nontuberculous mycobacterial (NTM) pulmonary disease are increasing worldwide, with *Mycobacterium avium* and *Mycobacterium abscessus* accounting for most infections (1, 2). NTM pulmonary disease involves multiple types of pulmonary lesions, including nodular bronchiectatic or fibrocavitary forms (1, 3), which frequently coexist and present with various clinical manifestations. From these, cavitation and severe bronchiectasis are generally associated with a poor prognosis (4), requiring the addition of parenteral amikacin to oral antimicrobial therapy (1). More recently, liposomal amikacin prepared for inhalation has been approved for patients with *M. avium* complex (MAC) pulmonary disease who fail to achieve culture conversion after at least 6 months of standard therapy (5). This highlighted the increasing importance of antimicrobial selection and dose optimization according to antimicrobial penetration characteristics into the disease phenotype.

Drug dose optimization is typically guided by pharmacokinetic–pharmacodynamic analysis based on plasma drug concentrations; however, plasma concentrations do not necessarily correlate with intrapulmonary concentrations (6). As anti-NTM pulmonary disease drugs are needed to act on the lung, intrapulmonary pharmacokinetics are important. However, as sampling from the lungs is more invasive and complex than plasma sampling, intrapulmonary pharmacokinetic data are limited. One study quantified the concentrations of first-line anti-tuberculosis agents in the epithelial lining fluid and alveolar cells in the lungs of 157 patients with tuberculosis (7). Although rifampicin did not show marked accumulation in the lungs relative to the plasma, ethambutol achieved substantially higher concentrations. However, this study evaluated only the lung-to-plasma concentration ratio of ethambutol and did not investigate parameters, such as the intrapulmonary half-life. Because both daily and three-times-weekly regimens are recommended for the treatment of NTM pulmonary disease (1), information on intrapulmonary half-lives is important for antimicrobial selection and dose optimization.

Another study has demonstrated that lesion type and heterogeneity influence drug penetration into lesions (8). Moreover, the extent of lesion penetration affects the sterilizing efficacy against mycobacterial populations there, as demonstrated in animal infection models (9–11). Strydom et al. (8) investigated lesion penetration of seven major antituberculosis drugs in 15 patients, finding that rifampicin and clofazimine exhibited comparable penetration into the cavity wall relative to a normal lung tissue. Notably, clofazimine showed low penetration into the large necrotic granuloma. However, neither study examined the penetration of azithromycin and amikacin, key drugs for NTM pulmonary disease, into lung lesions.

While intrapulmonary pharmacokinetics are relatively well-characterized for anti-tuberculosis drugs, such information remains very limited for anti-NTM pulmonary disease drugs. Furthermore, the penetration of antimicrobials used for NTM pulmonary disease into lung lesions remains poorly understood. Such information is important for selecting appropriate antimicrobials according to disease phenotype and optimizing dosing regimens. Therefore, this study aimed to characterize the intrapulmonary pharmacokinetics and lesion penetration of antimicrobials used to treat NTM pulmonary disease.

## RESULTS

Overall, 24 patients were enrolled, and their characteristics at surgery are summarized in Table 1. Reflecting the surgical indications of patients enrolled, a high proportion of patients presented cavitary disease (67%) and macrolide resistance (42%). The most frequently resected sites were the right middle lobe and left lingular segment. Detailed information on concomitant antimicrobial therapy, surgical sites, and resected lesion types for each patient is provided in Table S1. The median time from treatment initiation to surgery was 25 months (interquartile range, 5.8–34 months).

**TABLE 1:** Baseline characterization of study participants^*a*^

Characteristics	Value
Age (years)	60 ± 7.0
Female	22 (92%)
Body weight (kg)	52 ± 6.8
Body mass index (kg/m^2^)	20 ± 2.0
Causative bacterium	
*M. avium*	21 (88%)
*M. abscessus*	3 (13%)
Radiological findings	
Noncavitary—nodular bronchiectatic form	8 (33%)
Cavitary—nodular bronchiectatic form	14 (58%)
Fibrocavitary form	2 (8.3%)
Macrolide resistant	10 (42%)
Follow-up term (months)	28 [12–38]
Serum creatinine level (mg/dL)	0.71 ± 0.12
Estimated glomerular filtration rate (mL/min/1.73 m^2^)	69 ± 11
Creatinine clearance (mL/min)	73 ± 18

^*a*^ Mean ± standard deviation, Median (interquartile range), or Count (%).

**Fig 1:**
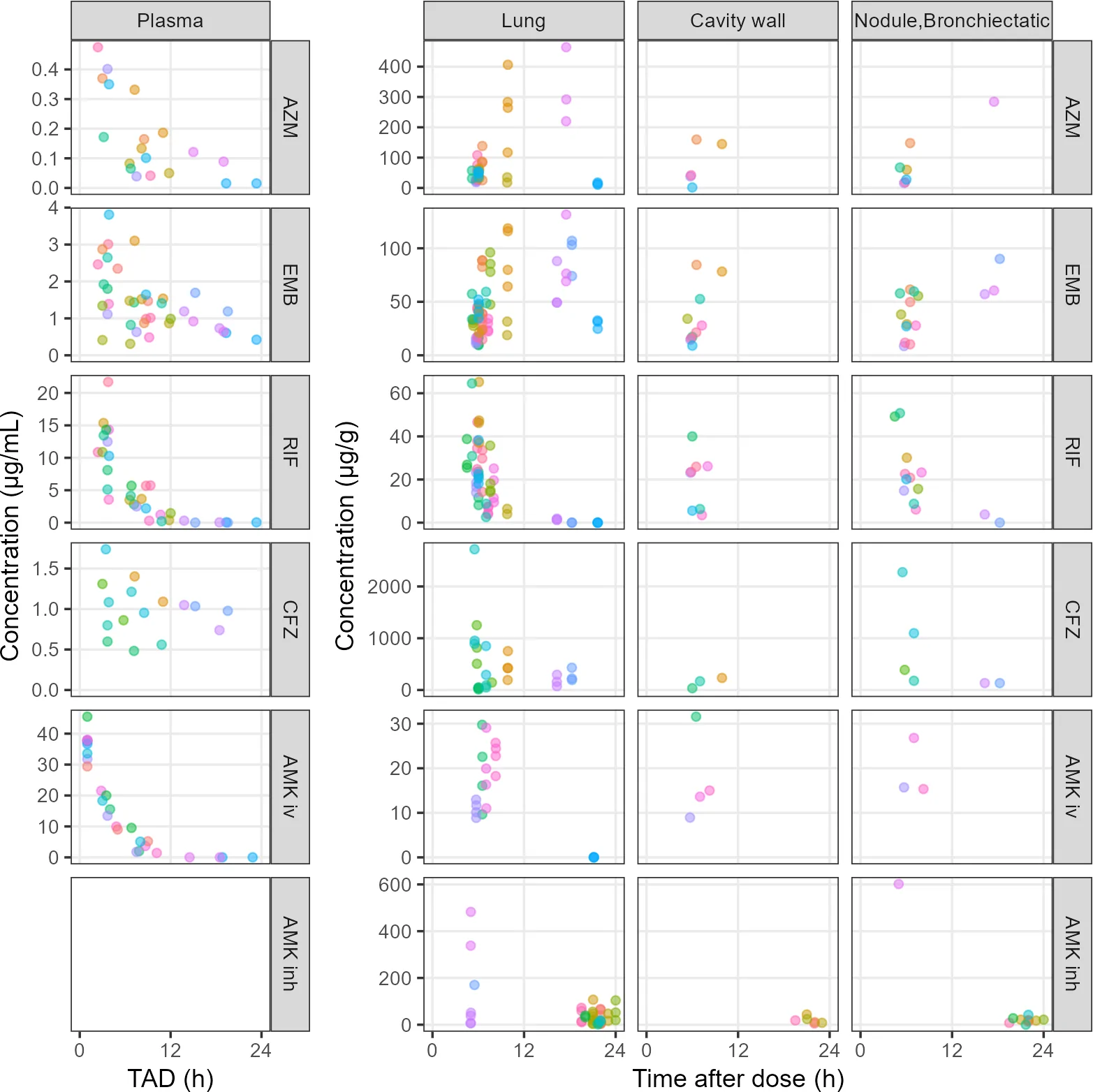
Concentration–time profiles of antimicrobial agents in plasma and lung tissues. Measured drug concentrations in plasma (μg/mL) and lung tissue (μg/g) are plotted against time after dosing for each patient (color-coded by individual). AZM, azithromycin; EMB, ethambutol; RIF, rifampicin; CFZ, clofazimine; AMK iv, intravenous amikacin; AMK inh, inhaled amikacin; TAD, time after dose.

Measured drug concentrations in plasma, normal lung tissue, and lesion sites are shown in Fig. 1. Two concentration measurements, one for rifampicin and the other for amikacin, fell below the lower limit of quantification and were excluded from subsequent analyses. Azithromycin, ethambutol, and clofazimine accumulated extensively in the lungs and were retained for prolonged periods.

**Fig 2:**
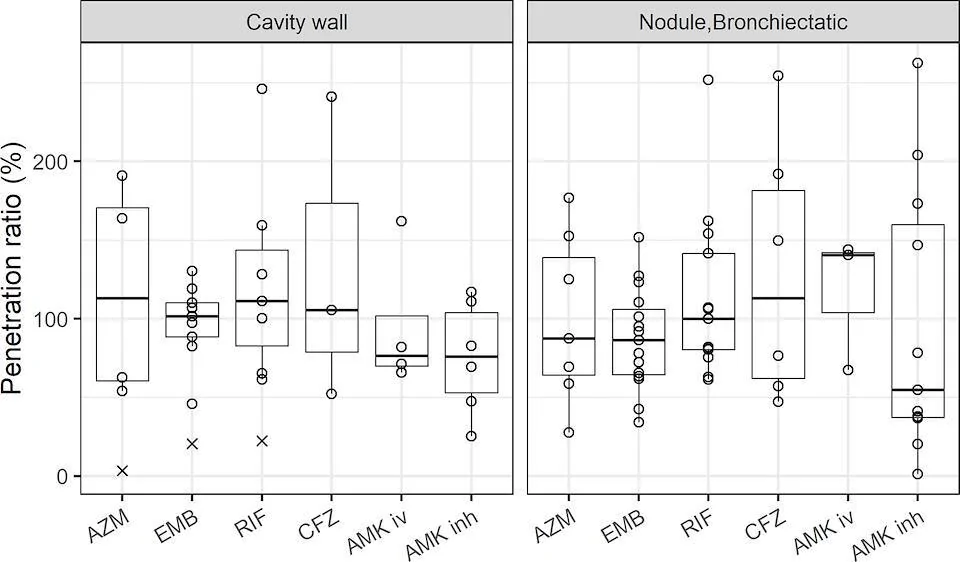
Drug penetration ratios into pulmonary lesions. Penetration ratio was calculated as the ratio of the lung lesion concentration divided by the mean concentration in a normal lung tissue of each patient, expressed as a percentage. A value of 100% indicates penetration equivalent to that of a normal lung tissue. Circles represent individual values, whereas crosses (×) represent the values obtained from the single caseum sample. AZM, azithromycin; EMB, ethambutol; RIF, rifampicin; CFZ, clofazimine; AMK iv, intravenous amikacin; AMK inh, inhaled amikacin.

None of the drugs showed lower penetration into the lesion sites (Fig. 2). However, drug concentrations in lung tissues exhibited substantial intra-individual variability, with coefficients of variation of 36, 25, 34, 47, 28, and 60% for azithromycin, ethambutol, rifampicin, clofazimine, intravenous amikacin, and inhaled amikacin, respectively, which was reflected in the high variability in penetration into pulmonary lesion sites. One sample obtained from a cavitary wall was identified as caseum, and the concentrations of azithromycin, ethambutol, and rifampicin in this sample were markedly lower than those in the normal lung tissue (Fig. 2).

**Fig 3:**
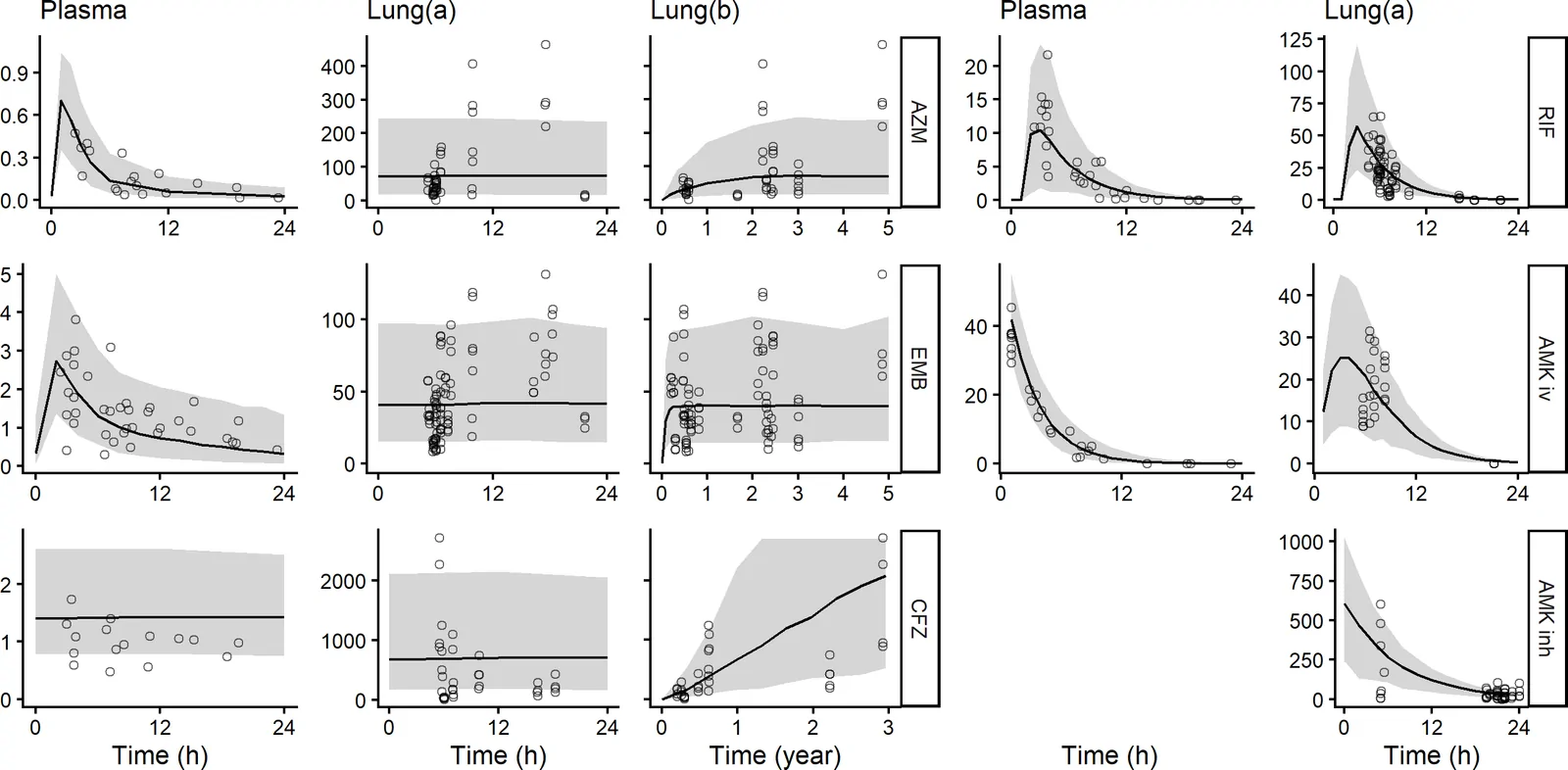
Model-predicted and measured drug concentration plots. Observed drug concentrations (circles) are plotted against model-predicted mean concentration–time profiles (solid lines), with shaded areas representing 90% prediction interval. For all drugs, the *y*-axis represents measured concentration (μg/mL for plasma; μg/g for the lung tissue), and the *x*-axis represents time after the last dose (h). For azithromycin, ethambutol, and clofazimine, which exhibited marked pulmonary accumulation, additional plots of lung concentration versus time since the first dose (years) are presented (lung [b]) to capture the full accumulation trajectory. AZM, azithromycin; EMB, ethambutol; RIF, rifampicin; CFZ, clofazimine; AMK iv, intravenous amikacin; AMK inh, inhaled amikacin.

Pharmacokinetic modeling was performed to estimate intrapulmonary pharmacokinetic parameters, such as intrapulmonary half-life, relevant to dosing optimization. For the plasma models, a two-compartment model best described the concentration profiles of azithromycin and ethambutol, whereas a one-compartment model provided the best fit for the other drugs. All tested covariate candidates were incorporated into the final models. In lung pharmacokinetic models, lesion type was evaluated as a candidate covariate for pulmonary partition coefficients; however, neither cavitary nor nodular/bronchiectatic lesions achieved significance. The goodness-of-fit plots (Fig. S1 to S5) and visual predictive checks (Fig. 3) demonstrated the robust predictiveness of the model. The model-predicted lung-to-plasma partition coefficients and intrapulmonary half-lives were of 535-fold and 6.3 months for azithromycin, 40-fold and 2.0 months for ethambutol, 5.2-fold and 6.3 min for rifampicin, 18800-fold and 22 years for clofazimine, and 1.3-fold and 2.3 h for intravenous amikacin, respectively. The estimated intrapulmonary half-life of inhaled amikacin exceeded that of intravenous amikacin by approximately 5 h. The detailed final model structures and parameter estimates are listed in Tables S2 to S6.

## DISCUSSION

This study characterizes the intrapulmonary pharmacokinetics of key antimicrobials used in NTM pulmonary disease. Azithromycin, ethambutol, and clofazimine showed lung concentrations more than 40-fold higher than blood concentrations, with observed lung half-lives exceeding 2 months. In contrast, rifampicin and amikacin showed lung concentrations only 1.3–5.2-fold higher than blood concentrations, and their lung half-lives were less than 5 h. Furthermore, the results suggested that all the drugs investigated showed similar penetration into lung lesions and normal lung tissue.

Ethambutol demonstrated marked pulmonary accumulation, with a lung-to-plasma concentration approximately 40-fold higher than the plasma concentration and a rifampicin ratio of 5.2-fold. These findings are broadly consistent with those of McCallum et al. (7) who reported plasma-to-lung concentration ratios in the alveolar cells of 15-fold for ethambutol and 1.4-fold for rifampicin. The quantitatively higher ratios observed in this study likely reflect methodological differences, as the homogenized lung tissue captures both intracellular and extracellular drug fractions, whereas isolated alveolar cell measurements do not. Ethambutol concentrations in the lung exhibited minimal intraday fluctuations, with an estimated intrapulmonary half-life of approximately 2 months, a novel finding with clinical importance. Thus, prolonged retention, even with intermittent dosing, may provide a mechanistic explanation for the suppression of macrolide resistance in *M. avium* pulmonary disease, even under a three-times-weekly regimen (12).

The population pharmacokinetic model predicted an average lung concentration of 76 μg/g for azithromycin. Previous studies have reported mean azithromycin concentrations in alveolar macrophages at steady state ranging from 40.4 to 205.2 μg/g (13, 14), which is comparable to concentration observed in this study. The observed lung half-life of azithromycin was 6 months, which was longer than the 2-month half-life observed for ethambutol. Therefore, if all antimicrobials were discontinued simultaneously, azithromycin could remain in the lungs as the sole antimicrobial between 2 and 6 months after discontinuation. This may potentially increase the risk of inducing macrolide resistance. Nevertheless, previous *in vitro* studies have reported that macrolides are less likely to develop resistance when their concentrations remain below the minimum inhibitory concentration and that the frequency of resistance emergence is lower when the bacterial burden is low (15). Therefore, this issue should be further investigated using simulations incorporating pharmacodynamics or prospective studies.

This is the first study to evaluate the intrapulmonary concentrations of clofazimine during long-term treatment. Clofazimine was orally administered for up to 3 years, during which time its concentration in the lungs continued to increase without reaching a plateau. Therefore, the relative standard error of lung half-life (22 years) estimated using the population pharmacokinetic model was 706%, suggesting an inherent imprecision of estimating this parameter (Table S5). Clofazimine is highly lipophilic and known to accumulate at high concentrations in various organs, with tissue deposition of crystallized clofazimine reported particularly during high-dose (≥300 mg/day) or long-term administration (16), which may explain the accumulation observed in this study.

The lung half-life of inhaled amikacin was longer than that following intravenous administration. This may be because, after inhalation, amikacin is initially deposited in the alveolar lining fluid and must subsequently distribute into lung cells and/or interstitial fluid before being transferred into systemic circulation. These processes may delay equilibration between the lung and systemic circulation, resulting in a longer apparent lung half-life following inhalation. Inhaled amikacin achieved a mean lung concentration of 26.9 μg/g (standard deviation 24.7) at approximately 20 h post-inhalation, broadly comparable to peak lung concentrations following intravenous administration. Siegel et al. (17) and Kurahara et al. (18) reported amikacin resistance rates of 18 and 15.9%, respectively, among patients with NTM pulmonary disease treated with amikacin liposome inhalation suspension. Sustained drug exposure in the lungs, while therapeutically desirable, may simultaneously contribute to resistance. In contrast to macrolides, the relationship between amikacin exposure and the development of amikacin resistance has not been well established. Moreover, as the inhaled amikacin used in this study was not a liposomal formulation, further investigation is required to clarify this association.

Penetration into cavitary and nodular/bronchiectatic lesions relative to the normal lung tissue was not markedly reduced for any of the tested drugs (Fig. 2), consistent with the findings of Strydom et al. (8) in tuberculosis, where rifampicin, clofazimine, and kanamycin exhibited penetration into the cavity wall comparable to normal lung tissues. Strydom et al. further demonstrated that clofazimine concentrations within the caseum were markedly lower than those in normal lungs, similar to what was observed in this study for azithromycin, ethambutol, and rifampicin, although in only one caseum sample (Fig. 2). These findings suggest that caseous lesions are less responsive to medical therapy.

This study has several limitations. First, the small sample size limited statistical power to detect differences in lesion penetration and precluded adequate characterization of the inter-individual variability in pulmonary drug concentrations. Second, the analysis was based on limited sampling time points, resulting in low precision of several population parameter estimates, which was evident for clofazimine, where lung concentrations did not plateau during the observation period. This resulted in relative standard errors of 676 and 706% for the lung partition coefficient and intrapulmonary half-life, respectively. Therefore, the temporal changes in tissue distribution during long-term therapy cannot be fully characterized, although the observation that clofazimine continues to accumulate in the lungs for at least 3 years is itself clinically relevant. Third, a direct relationship between the plasma and pulmonary concentrations and clinical outcomes, including treatment response and resistance suppression, has not been fully established. Fourth, the lung concentrations measured in this study could not distinguish between drug concentrations in intracellular and extracellular compartments, including the interstitial fluid. Therefore, the measured concentrations may not precisely reflect the drug concentrations to which the bacteria are exposed. Fifth, adherence to antimicrobial treatment was not monitored in the study participants. Finally, the potential influence of concomitant medications and patient-specific factors (e.g., disease severity and inflammatory status) could not be fully controlled. Additionally, residual confounding may have contributed to the observed variability in pulmonary drug concentrations.

Despite the limitations above, this study characterizes the intrapulmonary pharmacokinetics of antibiotics used for NTM pulmonary disease and provides important information for their optimizing dosing regimens. Furthermore, since none of the drugs clearly demonstrated reduced penetration into the lesion sites, lesion phenotype alone may have limited utility in guiding drug selection.

## MATERIALS AND METHODS

### Study design

This prospective, single-center, observational study was conducted at Fukujuji Hospital, Tokyo, Japan. This study was conducted in accordance with the ethical principles of the Declaration of Helsinki. The Institutional Review Boards of Fukujuji Hospital (no. 23018) and Meiji Pharmaceutical University (no. R5-004) approved the study. All participants provided written informed consent.

### Patient population

Patients aged ≥20 years who underwent guideline-based treatment for *M. avium* or *M. abscessus* pulmonary disease between November 2023 and February 2026 and were indicated for surgical intervention were enrolled in this study. Patients for whom dosing or administration times were unknown were excluded from this study. Considering the limited number of surgical cases of NTM pulmonary disease, sample size was not prespecified, and all patients enrolled during the study period were included in the analysis.

### Pharmacokinetic sampling

Antimicrobial agents were administered either on the morning of surgery or evening prior to surgery, and the administration times were recorded. Doses were administered according to guideline-recommended regimens (1): azithromycin 250 mg/day, ethambutol 10-15 mg/kg/day, rifampicin 10 mg/kg/day, clofazimine 100 mg/day, intravenous amikacin 10-15 mg/kg/day, and inhaled amikacin 600 mg/day. Owing to constraints of the Japanese healthcare reimbursement system, amikacin was administered as a nebulized intravenous formulation rather than an amikacin liposome inhalation suspension.

Blood samples were collected at the start and end of surgery. These were deproteinized using acetonitrile or 10% trichloroacetic acid, and drug concentrations were quantified using a validated high-performance liquid chromatography–mass spectrometry method (19).

Resected lung tissues were briefly rinsed with saline and subsequently dissected under a microscope to discriminate between the normal lung tissue and lesion sites (cavitary and nodular/bronchiectatic lesions). To characterize intra-individual variability in drug distribution within the normal lung tissue, normal lung parenchyma was sampled from four regions surrounding the lesion (superior, inferior, and bilateral), and each sample was analyzed separately. The entire lung tissue samples were homogenized, deproteinized, and quantified (19). Samples weighing less than 20 mg showed substantial variability in the measured concentrations and were, therefore, excluded from the analysis. The lower limits of quantification were 0.01 μg/mL for azithromycin, 0.02 μg/mL for ethambutol and clofazimine, 0.05 μg/mL for rifampicin, and 5 μg/mL for amikacin.

### Penetration ratios into lung lesions

For each patient, the average across the four normal lung tissue samples was calculated and used as the reference value. The lesion-to-normal concentration ratio was then computed for each drug and lesion type to determine pulmonary penetration.

### Pharmacokinetic modeling

Population pharmacokinetic models were developed using NONMEM (version 7.6.0; ICON Development Solutions, Hanover, MD, USA) following a two-stage approach. First, plasma-only pharmacokinetic models were developed independently for each drug. For intravenous amikacin, therapeutic drug monitoring data obtained within 1 week prior to surgery were also included in the model building. In the second stage, plasma and intrapulmonary pharmacokinetic data were analyzed using a tissue penetration model.

For the plasma models, one- and two-compartment models were tested. Since plasma concentration data during the absorption phase were unavailable, absorption-related pharmacokinetic parameters (absorption rate constant and lag time) could not be estimated and were fixed at values reported in the literature (20–24). Considering the sparse sampling design (two to three plasma measurements per individual), error was modeled using either an additive or proportional strategy, and inter-individual variability was estimated for clearance only. The base model was selected using the Akaike information criterion value, supported by relative standard error values and goodness-of-fit plots. Stepwise covariate modeling (25) was applied to identify significant covariates for the model (co-administration of rifampicin for azithromycin clearance and renal function assessed by Cockcroft–Gault estimated creatinine clearance or estimated glomerular filtration rate for ethambutol and amikacin clearance).

The lung-to-plasma partitioning of each drug was modeled as described by Anderson et al. (26) according to the following equation:


$$\frac{dC_{lung}}{dt}=k_{pl}\times \left(PC\times C_{plasma}-C_{lung}\right)$$

where *C* denotes drug concentration; *k_p_*_l_ is the plasma–lung equilibration rate constant (h^−1^); and *PC* is the partition coefficient representing the equilibrium ratio of lung-to-plasma drug concentration. Cavitary and nodular/bronchiectatic lesions were evaluated as candidate covariates for PC using a likelihood ratio test at a significance level of 5% (corresponding to a decrease in the objective function value ≥ 3.84).

## Data Availability

The data supporting the findings of this study are available from the corresponding author upon reasonable request.

## SUPPLEMENTAL MATERIAL

Supplemental material aac.00820-26-s0001.docx Tables S1 to S6; Fig. S1 to S5.

ASM does not own the copyrights to Supplemental Material that may be linked to, or accessed through, an article. The authors have granted ASM a non-exclusive, world-wide license to publish the Supplemental Material files. Please contact the corresponding author directly for reuse.
